# Development and validation of a real-time PCR assay for the detection of clinical acanthamoebae

**DOI:** 10.1186/s13104-017-2666-x

**Published:** 2017-07-28

**Authors:** Nessika Karsenti, Rachel Lau, Andrew Purssell, Ann Chong-Kit, Marlou Cunanan, Jason Gasgas, Jinfang Tian, Amanda Wang, Filip Ralevski, Andrea K. Boggild

**Affiliations:** 1 Department of Molecular Genetics and Microbiology, University of Toronto, Toronto, ON Canada; 2 Public Health Ontario Laboratories, Public Health Ontario, Toronto, ON Canada; 3 Faculty of Medicine, University of British Columbia, Vancouver, Canada; 4 Tropical Disease Unit, Division of Infectious Diseases, UHN-Toronto General Hospital, 200 Elizabeth St, 13EN-218, Toronto, ON M5G 2C4 Canada; 5 Department of Medicine, University of Toronto, Toronto, Canada

**Keywords:** Acanthamoebae, Amoebic keratitis, Corneal ulcer, Molecular diagnosis, Ophthalmology

## Abstract

**Background:**

Suboptimal agreement between molecular assays for the detection of *Acanthamoeba* spp. in clinical specimens has been demonstrated, and poor assay sensitivity directly imperils the vision of those affected by amoebic keratitis (AK) through delayed diagnosis. We sought to develop and validate a single Taqman real time PCR assay targeting the *Acanthamoeba* 18S rRNA gene that could be used to enhance sensitivity and specificity when paired with reference assays.

**Methods:**

Biobanked DNA from surplus delinked AK clinical specimens and 10 ATCC strains of *Acanthamoeba* was extracted. Sequence alignment of 66 18S rRNA regions from 12 species of *Acanthamoeba* known to cause keratitis informed design of a new TaqMan primer set. Performance of the new assay was compared to the 2 assays used currently in our laboratory.

**Results:**

Among 24 *Acanthamoeba*-positive and 83 negative specimens by the CDC reference standard, performance characteristics of the newly designed primer set were as follows: sensitivity 100%, specificity 94%, PPV 82.8%, and NPV 100%. Compared to culture, sensitivity of the new primer set was 100%, and specificity 96%. No cross-reactivity of the primer set to non-acanthamoebae, including *Balamuthia* and *Naegleria*, was found.

**Conclusions:**

We have validated a real time PCR assay for the diagnosis of AK, and in doing so, have overcome important barriers to rapid and sensitive detection of acanthamoebae, including limited sensitivity and specificity of commonly used assays.

**Electronic supplementary material:**

The online version of this article (doi:10.1186/s13104-017-2666-x) contains supplementary material, which is available to authorized users.

## Background

Species of the genus *Acanthamoeba* are known to cause amoebic keratitis (AK) among contact lens users [1]. AK due to environmental acanthamoebae manifests as corneal ulceration, which can lead to blindness if not treated in a timely and usually prolonged manner [1]. Other more easily detectable corneal pathogens, such as herpes simplex virus, cause a similar type of keratitis, and empiric treatment for these more common organisms leads to a delay in diagnosis of AK, which has a direct negative impact on patient outcomes. Increasing prevalence of AK coinciding the marketing and distribution of extended- or continuous-wear contact lenses [2] necessitates use of a high-performance detection method for known clinical and environmental strains of acanthamoebae [3].

Traditional diagnosis of AK has relied on historic parasitologic techniques such as culture, but while highly specific, this method is insensitive, inefficient, labor intensive, and requires technical expertise for interpretation. Molecular diagnostic techniques, such as polymerase chain reaction (PCR) performed directly on clinical specimens such as corneal scrapings and contact lens casings, are increasingly preferred over culture as they provide faster turnaround time (hours vs days) and eliminate the need for skilled microscopists. However, no single molecular assay has proven sufficiently sensitive for the range of known clinical and environmental acanthamoebae that can contaminate fresh water and contact lenses [4–7]. For clinical specimens, including corneal scrapings, contact lenses, and contact lens casings, individual PCR-based detection assays have reported sensitivities ranging from 64.3 to 93% compared to reference-level or composite diagnostics [4, 5], and specificities of 88.6–100% [4, 5]. Additionally, efficient clinical composite use of several assays in order to mitigate the risk of false negativity and positivity has been hindered by the validation of such assays on different diagnostic platforms: while some assays employ real time PCR (qPCR) [6, 7], others are used on an end-point platform [8, 9].

In our clinical reference laboratory, we routinely employ two molecular assays (the “Qvarnstrom” and “Riviere” assays; 6,7] as well as culture for the diagnosis of AK, however, this approach is time consuming, particularly when we need to arbitrate discordance with use of a third end-point PCR-based assay [5, 8]. The Riviere assay [6] was designed as a molecular diagnostic test specific to both trophozoites and cysts of *Acanthamoeba* spp. whereas the Qvarnstrom primer set [7] was designed as part of a multiplex assay detecting three free-living amoebae known to cause granulomatous amoebic encephalitis. While the Qvarnstrom assay covers a broader range of *Acanthamoeba* genotypes, the Riviere assay has a more sensitive limit of detection (LOD), with one study demonstrating that Riviere was able to detect as few as 10 DNA copies/μL, compared to the Qvarnstrom LOD of 43.8 DNA copies/μL [10]. Thus, in the interest of providing the most sensitive diagnostic approach for a disease caused by many species within the genus *Acanthamoeba* and one which typically produces an extremely low-volume clinical specimen, we have paired the two assays with culture.

We aimed to design a new real time PCR assay using a novel Taqman primer–probe set combining the excellent LOD of the Riviere assay with the species breadth of the Qvarnstrom assay in order to optimize our AK diagnostic workflow and service to our patient population.

## Methods

### Specimens

Delinked, surplus biobanked corneal scrapings and contact lenses and solutions stored at −80 °C after being processed for detection of acanthamoebae at the Public Health Ontario Laboratory between January 2012 and May of 2015 were identified and retrieved. In addition 10 ATCC strains of *Acanthamoeba*, as listed in Table 1, were also used for the validation. ATCC stains were cultured according to the recommended specifications, in peptone-yeast-glucose (PYG) medium [5].

**Table 1:** Reference standard positive ATCC specimens used in the validation of the new primer set, and species identification of positive clinical specimens

Source	Specimen	Species	Reference standard assay positive^a^
ATCC	ATCC50372	*A. polyphaga*	Both
	ATCC30173	*A. polyphaga*	Both
	ATCC50492	*A. polyphaga*	Both
	ATCC PRA-220	*Acanthamoeba* spp.	Both
	ATCC50372	*A. polyphaga*	Both
	ATCC50373	*A. castellanii*	Both
	ATCC50492	*A. castellanii*	Both
	ATCC50493	*A. castellanii*	Both
	ATCC50739	*A. castellanii*	Both
	ATCC30171	*A. culbertsoni*	Qvarnstrom
Clinical specimen	1	*A. castellanii*	Both
	2	*A. hatchetti*	Qvarnstrom
	3	*A. castellanii*	Both
	4	*A. castellanii*	Qvarnstrom
	5	Unknown	Qvarnstrom
	6	*A. polyphaga*	Both
	7	Unknown	Riviere
	8	*A. lenticulata*	Riviere
	9	*A. castellani*	Riviere
	10	*A. griffini*	Riviere
	11	Unknown	Riviere
	12	*A. castellanii*	Both
	13	*A. castellanii*	Both
	14	Unknown	Riviere
	15	*A. lenticulata*	Qvarnstrom
	16	Unknown	Riviere
	17	*A. hatchetti*	Both
	18	*A. polyphaga*	Both
	19	Unknown	Qvarnstrom
	20	Unknown	Qvarnstrom
	21	*A. quina*	Qvarnstrom

^a^Reference standard assays were Riviere [6] and Qvarnstrom [7], and a specimen was considered positive if either assay produced a logarithmic amplification curve with cycle threshold <40 on real time PCR

### Primer/probe design

66 18S DNA sequences from *Acanthamoeba* spp. were obtained from NCBI. These sequences include the same ones used by both Riviere [6] and Qvarnstrom [7] in the development of their primer sets, as well as 25 new sequences included in order to have at least one representative sequence for each species known to cause AK. Sequences were aligned using MEGA 6 software [11]. Aligned sequences were scanned visually for regions with a high occurrence of conserved nucleotides, and this region (bases 1946–2072 on the reference 18S rRNA sequence from the National Center for Biotechnology Information (NCBI), Accession Number AF019056) was used for Taqman MGB primer and probe design using Primer Express 3.0 software (Applied Biosystems, Foster City, CA). Primers and probe were subject to 2 BLAST searches each as a preliminary cross-reactivity check, one with *Acanthamoeba* species excluded, and one including only the human genome. Primers were also assayed for hairpin formation and degree of primer-dimer formation (Applied Biosystems, Foster City, CA).

### DNA extraction

DNA was extracted using the Qiagen Mini DNA extraction kit (Qiagen, Hilden, Germany. Samples were resuspended in PBS as needed, and subjected to three rounds of freeze–thaw in liquid nitrogen in order to lyse cysts before undergoing the manufacturer’s protocol. Elution was in 60 μL elution buffer to obtain more concentrated samples.

### DNA amplification

Each surplus clinical and ATCC sample was subject to qPCR using the newly designed primer and probe set, as well as the Riviere [6] and Qvarnstrom [7] primer sets, which served as our reference assays. The new primers used were AcanthF3 (5′-GGT CCG GGT AAT CTT TGC AAA-3′ TM = 60.6 °C), AcanthR2 (5′-GTA CAA AGG GCA GGG ACG TAA TCA AC-3′, TM = 66.2 °C) and the probe used is AcanthP2 (5′-[VIC]-TAAGCGCGAGTCATC-[MGB]-3′, TM = 70 °C). Taqman universal master mix was used according to the manufacturer’s protocol (ThermoFisher Scientific, Cat# 4304437, Waltham, MA), using 400 nM of each primer (AcanthF3 and AcanthR) and 200 nM of probe (AcanthP) for each reaction. qPCR was run using an initial cycle of 2 min at 50 °C, then 10 min at 95 °C and 45 cycles of 15 s at 95 °C and 1 min at 60 °C.

For the Riviere assay, 200 nM of forward (5′-CGA CCA GCG ATT AGG AGA CG-3′) and reverse (5′-CCG ACG CCA AGG ACG AC-3′) primers and 100 nM probe (5′-6-carboxyfluorescein [FAM]-TGA ATA CAA AAC ACC ACC ATC GGC GC-6-carboxytetramethylrhodamine [TAMRA]-3′) were used in each reaction [6]. For the Qvarnstrom assay, 400 nM of forward (5′-CCC AGA TCG TTT ACC GTG AA-3′) and reverse (5′-AAT ATT AAT GCC CCC AAC TAT CC-3′) primers and 200 nM probe (5′-FAM-CTG CCA CCG AAT ACA TTA GCA TGG-black hole quencher 1 [BHQ1]-3′) were used in each reaction [7]. TaqMan universal master mix (ThermoFisher Scientific, Cat# 4304437, Waltham, MA) was used according to manufacturer’s instructions, and 5 μl of DNA, in a total volume of 25 μl were run with the following cycling conditions: 50 °C for 2 min, 95 °C for 10 min, and 45 cycles of 95 °C for 15 s and then 60 °C (Riviere assay) or 55 °C (Qvarnstrom assay) for 1 min. All qPCR assays were run on an ABI 7500 real time PCR instrument (Applied Biosystems, Foster City, CA). Specimens were considered positive if the cycle threshold (Ct) value was <40 in the presence of a logarithmic amplification curve.

### Limit of detection determination

*Acanthamoeba* strains ATCC50373 and ATCC50739 were used to determine the limit of detection (LOD) of the new assay through serial dilutions of the DNA, from which a standard curve was generated. This standard curve was also used to calculate the efficiency of the assay according to the formula $E = 10^{{\left( {{1}\slash{s}} \right)}} - 1$, where S is the slope of the standard curve. Primers and probe were cross-checked against a variety of different keratitis-causing organisms and pathogenic eukaryotes (Table 2) in order to confirm lack of cross reactivity.

**Table 2:** Organisms used for cross-checking of primers to ensure lack of cross-species and genera reactivity

Organism	Source	Specimen type	Number of specimens analyzed
*Plasmodium falciparum*	Reference laboratory	Whole blood	4
*P. vivax*	Reference laboratory	Whole blood	5
*P. ovale*	Reference laboratory	Whole blood	3
*P. malariae*	Reference laboratory	Whole blood	1
*Entamoeba histolytica*	Reference laboratory	Stool	1
Herpes simplex 1	Reference laboratory	DNA from viral culture	1
Herpes simplex 2	Reference laboratory	DNA from viral culture	1
Rickettsiales	Reference laboratory	*Acanthamoeba* culture, endosymbiont	1
Chlamydiales	Reference laboratory	*Acanthamoeba* culture, endosymbiont	1
Legionellales	Reference laboratory	Clinical culture isolate	1

### Control human DNA

An important internal control used in our detection assay is the detection of human B2MG after spiking of samples post-extraction with human DNA to eliminate the possibility that a negative test result is due to local assay failure or PCR inhibitors. In order to determine if this spiking had an effect on the Ct value given by *Acanthamoeba*-specific primer sets, the 10 ATCC strains used in this validation were subject to qPCR as described above with the Riviere primer set both with and without human DNA added. The same was done with the new primer set. Due to limited specimen volumes, ATCC rather than clinical specimens were used for this experiment.

### Sequencing

Species identification of all positive clinical samples was performed in order to determine if the newly designed assay was sensitive enough to detect DNA from all represented species sent to our reference laboratory. Amplification of DNA and sequencing was performed on each sample using three different primers. The Nelson primers [9], JDP primers [8] (JDP1 and JDP2), and a newly developed set of end point PCR primers made specifically for sequencing of the 18S rRNA region of *Acanthamoeba* were used. The newly designed sequencing primer set was developed to bind to a conserved region for all of the different species and to allow the amplicon to span as much of the gene as possible, in order to sequence across the heterogeneous regions to differentiate species. The AcanSeqF (5′-CCT ACC ATG GTC GTA ACG GG-3; TM = 64.5 °C) and AcanSeqR (5′-AGG GCA GGG ACG TAA TCA AC-3′, TM = 62.5 °C) primers were designed for this purpose. Sanger sequencing was performed on these samples using BigDye v3.1 (ThermoFisher Scientific, Waltham, MA) and a 3130 × l genetic analyzer according to the standard protocol. Gathering data obtained from all 3 primer sets and aligning them with Contig express (Invitrogen, Carlsbad, CA) enabled greater confidence in the bases identified, and allowed improved confidence in species identification. Obtained sequences were subject to a BLAST search in order to determine the species with the highest homology to the specimen.

### Statistical analysis

Data were managed in a password-protected Microsoft Excel file (Redmond, WA). Primary outcome measures were sensitivity, specificity, positive predictive value (PPV), and negative predictive value (NPV), and were calculated in the standard manner using a reference standard of the Qvarnstrom (CDC) assay. Confidence intervals for these performance metrics were determined using Vassarstats Clinical Calculator 1 (http://vassarstats.net/clin1.html). Secondary outcome measures were performance characteristics compared to the composite reference standard of either Qvarnstrom [7] or Riviere [6] assay positivity, as well as compared to clinical culture. Mean Ct values were compared by t test for both the new assay and Riviere with and without addition of human control DNA using GraphPad Prism v. 7.0 (GraphPad Inc., La Jolla, CA).

## Results

Between January 2012 and May 2015, 97 surplus specimens submitted for *Acanthamoeba* diagnostics were biobanked as follows: corneal scrapings (n = 87), contact lenses in saline (n = 4) or contact lens solution (n = 6). Twenty-one clinical specimens (21.6%) were known to be positive using a combination of qPCR and culture, and these served as our reference standard positives for the primary and secondary outcomes analysis, along with the 10 ATCC strains of *Acanthamoeba*. Seventy-six clinical specimens (78.4%) were considered true negatives.

### Assay performance characteristics compared to Qvarnstrom [7] reference standard

Of 107 total specimens (97 banked and 10 ATCC specimens), 29 (27.1%) were positive (10/10 ATCC strains and 19/97 clinical specimens) and 78 (72.9%) were negative (0/10 ATCC strains and 78/97 clinical specimens) using the new primer set, corresponding to a sensitivity of 100% (95% CI 82.8–100%), specificity of 94.0% (95% CI 85.9–97.8%), PPV of 82.8%, and NPV of 100% compared to the Qvarnstrom reference assay [7], where 24 specimens were positive and 83 specimens were negative (Table 3; 2 × 2 table available as a Additional files 1, 2). All 10 ATCC strains of *Acanthamoeba* were detected by the new primer set.

**Table 3:** Performance characteristics of the new assay compared to the Qvarnstrom assay [7]

Primer Set	Positive (N)	Negative (N)	Sensitivity (%)	Specificity (%)	PPV (%)	NPV (%)
New primer set	29	78	100% (95% CI 82.8–100%)	94.0% (95% CI 85.9–97.8%)	82.8	100

By Qvarnstrom, 24 specimens were considered true positive, and 83 were considered true negative

### Assay performance characteristics compared to composite reference standard

Of 107 total specimens, 31 (29%) were positive (10/10 ATCC strains and 21/97 clinical specimens) by either or both of the Qvarnstrom and Riviere assays, and 76 (71%) were negative (0/10 ATCC strains and 76/97 clinical specimens) by both reference assays. Performance characteristics of the new primer set compared to the composite of either the Qvarnstrom or Riviere assays were as follows: sensitivity 93.5% (95% CI 77.2–98.9%), specificity of 100% (95% CI 94.0–100%), PPV 89.7%, and NPV 93.6% (Table 4; 2 × 2 table available as a Additional files 1, 2). Three of 5 (60%) false negative specimens (i.e., positive by only the Riviere assay) could not be species identified by our sequencing methodology. One false negative (by both Qvarnstrom and the new primer set), was identified by sequencing as *A. griffini* and another as *A. castellani*. Ct values for the 5 false negative specimens (detected by the Riviere assay only) were higher than average (36–39 vs 31).

**Table 4:** Performance characteristics of the new assay compared to the composite reference standard of either or both Qvarnstrom [7] and Riviere [6] assay positivity

Primer Set	Positive (N)	Negative (N)	Sensitivity (%)	Specificity (%)	PPV (%)	NPV (%)
New primer set	29	78	93.5% (95% CI 77.2–98.9%)	100% (95% CI 94.0–100%)	100	97.4

By either or both of Qvarnstrom and Riviere, 31 specimens were considered true positive, and 76 were considered true negative

### Assay performance characteristics compared to culture reference standard

Of 107 specimens, 37 (34.6%) underwent culture due to availability of clinical specimen: 12 (32.4%) were culture positive (10/10 ATCC strains and 2/27 clinical specimens), and 25 (67.6%) were culture negative (0/10 ATCC strains and 25/27 clinical specimens). Compared to culture, the new primer set demonstrated the following performance characteristics: sensitivity 100% (95% CI 69.9–100%), specificity 96.0% (95% CI 77.7–99.8%), PPV 92.3%, and NPV 100% (Table 5; 2 × 2 table available as a Additional files 1, 2). Both the new assay and Qvarnstrom assay detected all acanthamoebae from ATCC and clinical specimens that were culture positive, unlike the Riviere assay, which failed to detect acanthamoebae in 2 ATCC and clinical specimens that were culture positive.

**Table 5:** Performance characteristics of the new assay, as well as Riviere [6] and Qvarnstrom [7] compared to culture as the reference standard

Primer Set	Positive (N)	Negative (N)	Sensitivity (%)	Specificity (%)	PPV (%)	NPV (%)
New primer set	13	24	100% (95% CI 69.9–100%)	96.0% (95% CI 77.7–99.8%)	92.3	100
Riviere assay [6]	11	26	91.7% (95% CI 59.8–99.6%)	100% (95% CI 83.4–100%)	100	96.1
Qvarnstrom assay [7]	12	25	100% (95% CI 69.9–100%)	100% (95% CI 83.4–100%)	100	100

By culture, 12 specimens were considered true positive and 25 specimens were considered true negative

### Limit of detection

LOD of the new assay was calculated to be 7 copies/μL using ATCC50739, with an efficiency of 89.2%. Using ATCC50373, the strain used as a positive control for all experimentation, a LOD of 11 copies/μL was obtained, with an efficiency of 130%. Standard curves constructed from serial dilutions of both strains are shown in Additional file 1: Figure S1.

### Assay cross-reactivity

BLAST searches using the primers and probe as targets showed only 18S rRNA of *Acanthamoeba* when left unfiltered; excluding *Acanthamoeba* or including only the human genome revealed no possibility for cross-reactivity (data not shown). Cross-checking of the primer set with 13 other microbial species revealed lack of cross-reactivity with any other amoeba, protist, or microbe typically found in the eye, or bacterium found to be a natural endosymbiont of *Acanthamoeba* (Table 2).

### Human DNA

The effect of adding human DNA to primary clinical samples as a local assay control was analyzed as described above. On average, Ct values did not change with addition of human DNA when the Riviere primer set was used (average increase = 0.1, p = 0.774). However, Ct values increased by 3.8 with the new primer set (p = 0.0013) (Additional file 1: Figure S2).

### Species identification

Of 21 positive surplus clinical specimens included in this validation, we were able to species identify 14 (66.7%) as follows: *A. castellanii* (n = 6), *A. hatchetti* (n = 2), *A. polyphaga* (n = 2), *A. lenticulata* (n = 2), *A. quina* (n = 1), and *A. griffini* (n = 1). The remaining 8 reference positive specimens could not be species identified using our sequencing method. Specimens that could not be species identified all showed higher Ct values by both the Qvarnstrom and Riviere primer sets (data not shown), potentially indicating less DNA in the clinical sample.

## Discussion

AK is a potentially blinding ocular infection caused by environmentally ubiquitous acanthamoebae, whose varied adaptations to specific niches and opportunistic proclivities, as well as genetic heterogeneity challenge the development of both highly sensitive and specific molecular assays for their clinical detection. We have previously demonstrated that the Riviere assay, which has an excellent LOD, failed to detect specific clinically relevant strains of *Acanthamoeba* [5]. Due to the typically minuscule volume of specimen attainable from the cornea of patients with AK, any molecular assay implemented clinically for acanthamoebae detection must demonstrate, in addition to broad species sensitivity, a very low LOD. We developed a Taqman-based assay for the molecular detection of acanthamoebae from common clinical specimens such as corneal scrapings and contact lenses with the aim of combining excellent species coverage with analytic sensitivity.

### Targeting the 18S rRNA gene is both sensitive and specific

The 18S rRNA gene is commonly used for developing primers for both detection and sequencing of protists because of its high conservation across the target genus, and its regions of variation that can be used to distinguish species. Furthermore the 18S region is part of a ribosomal tandem repeat segment, meaning it is present in multiple copies across the genome. Some estimates place the 18S copy number per organism for *Acanthamoeba* at around 60 [12]. Mathers and colleagues [9] designed a PCR primer set (“Nelson”) based on the 18S rRNA region of the genome to detect *Acanthamoeba* in clinical samples, while Schroeder and colleagues [8] developed a second primer set, the “JDP” set, that amplified a larger region of the 18S rRNA gene of *Acanthamoeba*. We have previously demonstrated that, using clinical corneal specimens, the Nelson primer set was more sensitive (90% vs 65%), whereas the JDP primer set was more specific (100% vs 90.8%) [4]. Thus, neither assay could stand alone for the diagnosis of AK in a clinical laboratory. The Riviere primer set [6] was designed by aligning sequences of 6 different 18S rRNA genomes, thus, while sensitive from a LOD perspective, the assay appears to be unable to detect the broad range of *Acanthamoeba* species and genotypes known to cause AK, as previously demonstrated [5, 10, 13].

The new primer set, based on alignment of 66 different 18S *Acanthamoeba* sequences, demonstrated sensitivity of 100% compared to reference assays such as culture and the CDC’s Qvarnstrom [7] assay, with specificities of 96 and 94%, respectively. Although the increase in Ct values with addition of human control DNA for the new primer set could potentially lead to false negativity, it should be noted that even with a 4-cycle increase in Ct value, all positive specimens remained in the detectable range (<40 cutoff). Species identification was possible for just over 60% of clinical specimens containing acanthamoebae. The new assay demonstrated broad species detection, but was unable to detect a strain of *A. griffini* and *A. castellani*, which were detected only by the Riviere assay. Conversely, the new assay detected *Acanthamoeba* DNA in both clinical and ATCC strains of *A. culbertsoni* and *A. lenticulata*, both of which are known to produce false negative results using the Riviere assay alone [5, 10, 13].

### Pairing of assays is optimal for diagnostic workflow

The new primer set is run at a 60 °C elongation temperature, which is the same temperature used by the Riviere assay [6]. Therefore, these two assays can be run in parallel on the same plate to potentially increase sensitivity of the new assay. Because the Riviere assay has a LOD of 10 and efficiency of 91% [6], it has high analytic sensitivity. Due to limited clinical sensitivity of Riviere compared to Qvarnstrom, however, the new assay would augment the range of genotypes and species detectable in clinical specimens [4, 5, 10, 13]. As we were unable to species identify 60% of the specimens detected only by the Riviere assay, the ‘trueness’ of their positivity is unknown, particularly in light of the late Ct values noted for those specimens. Measures of sensitivity, specificity, PPV, NPV, LOD or efficiency are unavailable in the Qvarnstrom paper [7], and as such, we cannot directly compare these performance metrics. In addition, the Qvarnstrom assay is run with an elongation temperature of 55 °C [7], so running the new assay in parallel with Qvarnstrom would require preparation of separate plates, and consequent reduction in workflow efficiency. Our data indicate that all clinical specimens deemed positive by the Qvarnstrom assay were also detected by the new primer set.

### Limitations

The new assay failed to detect acanthamoebae in 5 clinical specimens deemed positive by the Riviere assay, and because 3 of these organisms could not be identified to the species level, we are unable to draw conclusions about potential species or genotypes missed by the new assay. However, both the inability to species identify them through sequencing and the inability of the new assay to detect them could point to a low DNA content in the sample itself. Second, due to limited volumes of clinical specimens, only 37 during the enrolment period were set-up for culture, and this may have introduced bias to our findings and interpretation. Finally, our use of surplus banked clinical specimens may have led to under-representation of positives due to exhaustion of acanthamoebae from primary specimens during initial clinical testing. Better precision of the performance characteristics can be ascertained when this new assay is implemented on all clinical samples received prospectively.

## Conclusions

Rapid and accurate detection of AK is imperative to timely, definitive treatment, and preservation of vision. We have designed and validated a new Taqman-based assay targeting the 18S rRNA region of *Acanthamoeba*, which demonstrated excellent clinical sensitivity and specificity compared to reference assays such as culture and the CDC’s Qvarnstrom assay [7], as well as excellent analytic sensitivity. As labour-intensive and specialized *Acanthamoeba* culture offered no diagnostic advantage over the new assay, this component of our current diagnostic algorithm, which has a prolonged turnaround time, can likely be discarded without sacrificing either clinical sensitivity or specificity.

## Additional files

**Additional file 1: Figure S1.** Standard curves used for calculating limit of detection (LOD) of the primer set, comprised of Acanth-F3, Acanth-R2 and Acanth-P2. Two curves are shown, using ATCC strains of Acanthamoeba castellanii (ATCC50373 and ATCC50739). **Figure S2.** Comparison of Ct values with and without human DNA added for detection of human B2MG as a control. Samples with and without human DNA added were analyzed by both the Riviere (6) assay and the new primer set to determine the effect of human DNA on Ct value. Ct values were higher using the new primer set following addition of control human DNA (p = 0.0013), but not using the Riviere assay (p = 0.744).

**Additional file 2: Table S1.** 2 × 2 table for calculating performance characteristics of the new diagnostic assay compared to the Qvarnstrom (1) assay. **Table S2.** 2 × 2 table for calculating performance characteristics of the new diagnostic assay compared to the composite reference standard of either or both the Qvarnstrom (1) and Riviere (2) assays. **Table S3**. 2 × 2 table for calculating performance characteristics of the new diagnostic assay compared to the reference standard of culture.

## Abbreviations

AK: amoebic keratitis
ATCC: American Type Culture Collection
CDC: Centers for Disease Control and Prevention
Ct: cycle threshold
FAM: 6-carboxyfluorescein
LOD: limit of detection
NCBI: National Center for Biotechnology Information
NPV: negative predictive value
PPV: positive predictive value
PYG: peptone-yeast-glucose
qPCR: real time PCR
TAMRA: 6-carboxytetramethylrhodamine
