# Supplementary material for: Development and validation of a real-time PCR assay for the detection of clinical acanthamoebae
Source: BMC Res Notes. 2017 Jul 28;10:355. doi: 10.1186/s13104-017-2666-x (PMC5534048; doi:10.1186/s13104-017-2666-x)

**Development and Validation of a Real-time PCR Assay for the Detection of Clinical Acanthamoebae**

Supplementary File

Supplementary Figure 1. Standard curves used for calculating limit of detection (LOD) of the primer set, comprised of Acanth-F3, Acanth-R2 and Acanth-P2. Two curves are shown, using ATCC strains of *Acanthamoeba castellanii* (ATCC50373 and ATCC50739).


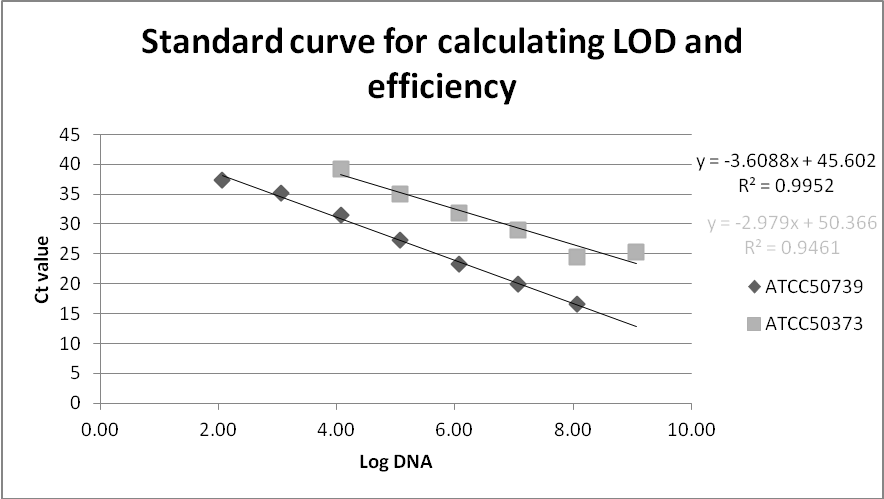


Supplementary Figure 2. Comparison of Ct values with and without human DNA added for detection of human B2MG as a control. Samples with and without human DNA added were analyzed by both the Riviere (6) assay and the new primer set to determine the effect of human DNA on Ct value. Ct values were higher using the new primer set following addition of control human DNA (p=0.0013), but not using the Riviere assay (p=0.744).


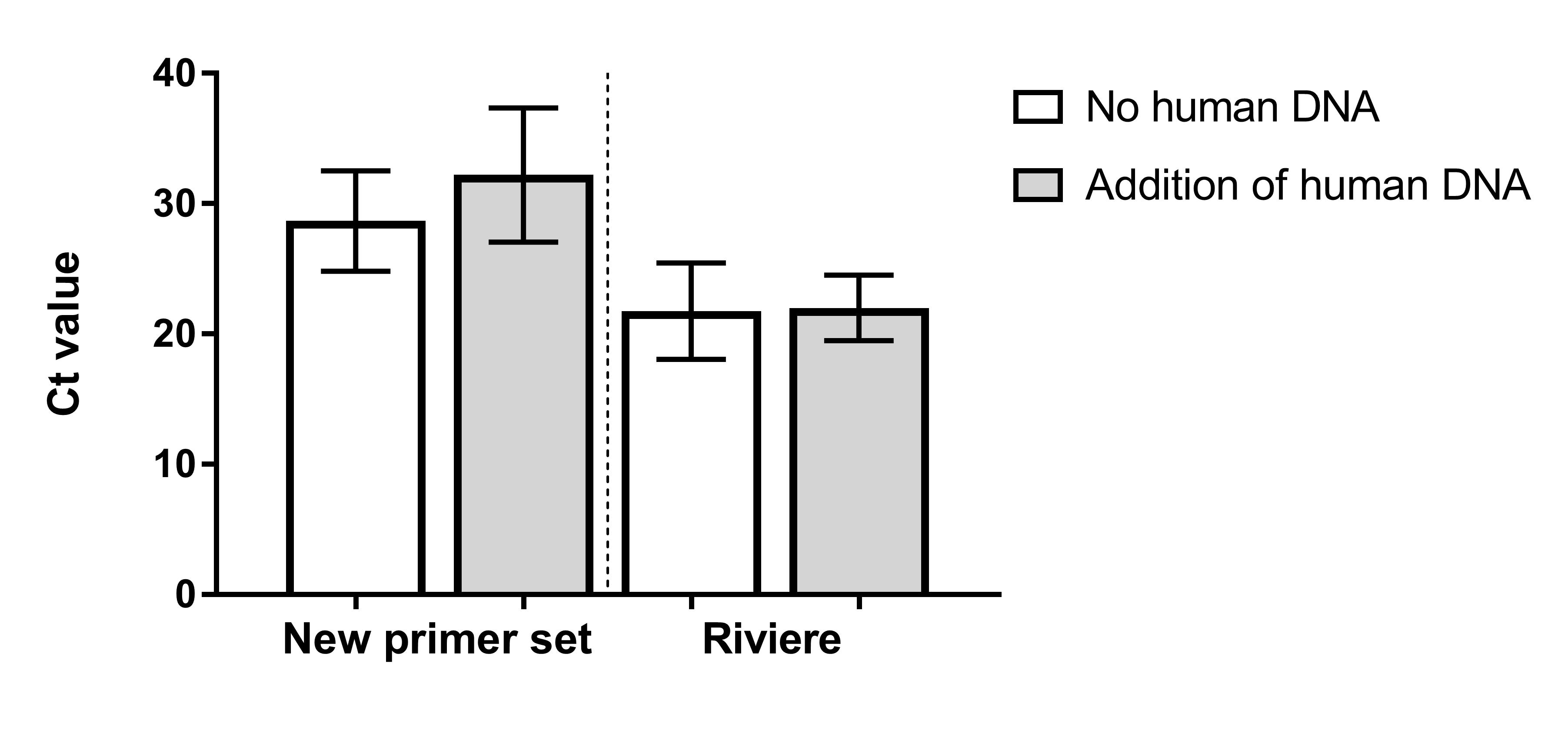

Supplement: Supplementary file 1 — Additional file 1: Figure S1. Standard curves used for calculating limit of detection (LOD) of the primer set, comprised of Acanth-F3, Acanth-R2 and Acanth-P2. Two curves are shown, using ATCC strains of Acanthamoeba castellanii (ATCC50373 and ATCC50739). Figure S2. Comparison of Ct values with and without human DNA added for detection of human B2MG as a control. Samples with and without human DNA added were analyzed by both the Riviere (6) assay and the new primer set to determine the effect of human DNA on Ct value. Ct values were higher using the new primer set following addition of control human DNA (p = 0.0013), but not using the Riviere assay (p = 0.744). [file 13104_2017_2666_MOESM1_ESM.doc]
