# Supplementary material for: Development and validation of a real-time PCR assay for the detection of clinical acanthamoebae
Source: BMC Res Notes. 2017 Jul 28;10:355. doi: 10.1186/s13104-017-2666-x (PMC5534048; doi:10.1186/s13104-017-2666-x)
Supplement: Supplementary file 2 — Additional file 2: Table S1. 2 × 2 table for calculating performance characteristics of the new diagnostic assay compared to the Qvarnstrom (1) assay. Table S2. 2 × 2 table for calculating performance characteristics of the new diagnostic assay compared to the composite reference standard of either or both the Qvarnstrom (1) and Riviere (2) assays. Table S3. 2 × 2 table for calculating performance characteristics of the new diagnostic assay compared to the reference standard of culture. [file 13104_2017_2666_MOESM2_ESM.doc]

**Development and Validation of a Real-time PCR Assay for the Detection of Clinical Acanthamoebae**

Supplementary File.

2 x 2 table for calculating performance characteristics of the new diagnostic assay compared to the Qvarnstrom (1) assay

|  | | **New Primers** | | Total |
| --- | --- | --- | --- | --- |
|  |  | Positive | Negative |  |
| **Gold Standard (Qvarnstrom [1] positive)** | Positive | 24 | 0 | 24 |
|  | Negative | 5 | 78 | 83 |
| **Total** | | 29 | 78 | 107 specimens |

2 x 2 table for calculating performance characteristics of the new diagnostic assay compared to the composite reference standard of either or both the Qvarnstrom (1) and Riviere (2) assays

|  | | **New Primers** | | Total |
| --- | --- | --- | --- | --- |
|  |  | Positive | Negative |  |
| **Gold Standard (Riviere [2] or Qvarnstrom [1] positive)** | Positive | 26 | 5 | 31 |
|  | Negative | 3 | 73 | 76 |
| **Total** | | 29 | 78 | 107 specimens |

2 x 2 table for calculating performance characteristics of the new diagnostic assay compared to the reference standard of culture

|  | | **New Primers** | | Total |
| --- | --- | --- | --- | --- |
|  |  | Positive | Negative |  |
| **Gold Standard Culture** | Positive | 12 | 0 | 12 |
|  | Negative | 1 | 24 | 25 |
| **Total** | | 13 | 24 | 37 specimens |

References:

1. Qvarnstrom, Y., Visvesvara, G., Sriram, R., & Silva, A. (2006). Multiplex Real-Time PCR Assay for Simultaneous Detection of Acanthamoeba spp., Balamuthia mandrillaris, and Naegleria fowleri. *Journal of Clinical Microbiology,* *44*(10), 3589-3595.
2. Rivière, D., Szczebara, F., Berjeaud, J., Frère, J., & Héchard, Y. (2005). Development of a real-time PCR assay for quantification of Acanthamoeba trophozoites and cysts. *Journal of Microbiological Methods,* *64*, 78-83.
